# Differential Effects of Nasal Inflammation and Odor Deprivation on Layer-Specific Degeneration of the Mouse Olfactory Bulb

**DOI:** 10.1523/ENEURO.0403-19.2020

**Published:** 2020-04-02

**Authors:** Sanae Hasegawa-Ishii, Fumiaki Imamura, Shin Nagayama, Makiko Murata, Atsuyoshi Shimada

**Affiliations:** 1 Pathology Research Team, Faculty of Health Sciences, Kyorin University, Mitaka, Tokyo 181-8612, Japan; 2 Department of Pharmacology, Penn State College of Medicine, Hershey, PA 17033-0850; 3 Department of Neurobiology and Anatomy, McGovern Medical School at the University of Texas Health Science Center at Houston, Houston, TX 00730-1501

**Keywords:** atrophy, nasal inflammation, neuro-inflammation, odor deprivation, olfactory bulb, olfactory system

## Abstract

Harmful environmental agents cause nasal inflammation, and chronic nasal inflammation induces a loss of olfactory sensory neurons (OSNs) and reversible atrophy of the olfactory bulb (OB). Here, we investigated the mechanisms underlying this inflammation-induced OB atrophy by histologically and biochemically comparing the OB changes in mouse models of nasal inflammation and odor deprivation. In addition, we examined whether odor stimulation is necessary for OB recovery from atrophy. One group of adult male C57BL/6 mice was administered lipopolysaccharide (LPS) unilaterally for 10 weeks to induce nasal inflammation (control animals received saline), and a second group received unilateral naris closures (NCs) for 10 weeks of odor deprivation. The OBs atrophied in both models, but odor deprivation shrank the glomerular, external plexiform, mitral, and granule cell layers (GCLs), whereas the olfactory nerve, glomerular, and external plexiform layers (EPLs) atrophied as a result of nasal inflammation. Additionally, nasal inflammation, but not odor deprivation, caused neuroinflammation in the OB, inducing glial activation and elevated expression of interleukin-1β (IL-1β) and TNFα. After 10 weeks of nasal inflammation, mice were housed for another 10 weeks with no additional treatment or with unilateral NC. Nasal inflammation and glial activation subsided in both groups, but glomerular and EPLs recovered only in those with no additional treatment. Our findings demonstrate that nasal inflammation and odor deprivation differentially induce layer-specific degeneration in the OB, that loss of OSN activity rather than neuroinflammation is a major cause of inflammation-induced OB atrophy, and that odor stimulation is required for OB recovery from atrophy.

## Significance Statement

Chronic nasal inflammation causes a loss of olfactory sensory neurons (OSNs) and atrophy of the olfactory bulb (OB), which recovers when inflammation subsides. To reveal the mechanisms underlying inflammation-induced OB atrophy, we compared the histologic and biochemical changes in OBs in mouse models of nasal inflammation and odor deprivation. In addition, we examined whether odor stimulation was required for the recovery of the OB from atrophy. Our findings revealed that nasal inflammation and odor deprivation differentially induce layer-specific degeneration in the OB, that loss of OSN activity rather than neuroinflammation is a major cause of inflammation-induced OB atrophy, and that odor stimulation is required for the OB to recover from atrophy.

## Introduction

Rhinitis, sinusitis, and rhinosinusitis are common diseases often caused by exposure to environmental allergens, such as bacteria, viruses, molds, and pollens (Ahmad and Zacharek, 2008; Imamura and Hasegawa-Ishii, 2016). A major symptom of these diseases is nasal inflammation that causes nasal congestion and/or a runny nose. However, nasal inflammation is also associated with many neurologic disorders. For example, anxiety and depression are more prevalent in patients with rhinitis than in healthy subjects (Kim et al., 2016; Schlosser et al., 2016; Bedolla-Barajas et al., 2017). Moreover, olfactory dysfunction, as well as a runny nose, is a common and early sign of neurodegenerative diseases such as Parkinson’s and Alzheimer’s diseases (Chou et al., 2011; Doty, 2012; Daulatzai, 2015), and patients with depression exhibit reduced olfactory performance (Yuan and Slotnick, 2014; Siopi et al., 2016; Rochet et al., 2018). However, the mechanisms connecting nasal inflammation with neurologic disorders are still an enigma. As agents and factors in the nasal passage can bypass the blood-brain barrier (Doty, 2008; Dando et al., 2014), it is important to understand how nasal inflammation affects brain structure and functions.

Chronic rhinosinusitis, as well as Parkinson’s disease and depression, has been linked with reduced olfactory bulb (OB) volume (Rombaux et al., 2008; Negoias et al., 2010; Li et al., 2016; Rottstädt et al., 2018). A previous study showed that chronic nasal inflammation induced by intranasal lipopolysaccharide (LPS) administration causes glial activation and a loss of synapses in the OB within three weeks (Hasegawa-Ishii et al., 2017). Prolonged inflammation (10 weeks or more) results in atrophy of the OB, which recovers once the inflammation resolves (Hasegawa-Ishii et al., 2019). The atrophy is attributed to the shrinkage of superficial layers of the OB, namely, the olfactory nerve layer (ONL), glomerular layer (GL), and external plexiform layer (EPL), especially the superficial EPL (sEPL; Hasegawa-Ishii et al., 2019). The shrinkage of the ONL and GL results from the loss of olfactory sensory neuron (OSN) axons and damage to the olfactory epithelium (OE) from persistent nasal inflammation. However, the reason for the loss of the EPL is largely unknown, because OSN axons do not penetrate this layer comprising the secondary dendrites of projection neurons, including mitral and tufted cells, which synapse with granule cell dendrites. Further studies are needed to determine the mechanism of EPL shrinkage and recovery from nasal inflammation-induced atrophy to better understand the roles of the OB and olfactory cortex in nasal inflammation-associated brain damage.

Previous studies also showed that intranasal LPS administration induced gliosis in the EPL, which may have contributed to EPL shrinkage (Hasegawa-Ishii et al., 2017, 2019). On the other hand, the loss of odor input as a result of OSN loss may also have contributed. Long-term odor deprivation via unilateral naris closure (NC) induces OB atrophy and decreases the volume of the EPL and granule cell layer (GCL), with no evidence of histologic changes in the OE (Henegar and Maruniak, 1991; Maruniak et al., 1989a,b, 1990). However, it is not known whether NC induces inflammatory responses and which part of the EPL is affected by odor deprivation. Therefore, we performed a detailed examination of the histologic and biochemical changes and inflammatory responses in the OB and OE in adult mice experiencing long-term odor deprivation and chronic nasal inflammation.

## Materials and Methods

### Animals

Eight-week-old male C57BL/6JJmsSlc mice (Sankyo lab) were used in this study. The mice were deeply anesthetized with isoflurane and intranasally administered 10-μl physiologic saline or LPS from *Escherichia coli* O55:B5 (1 mg/ml; Sigma) three times per week for three, six, or 10 weeks (saline:3w, saline:6w, and saline:10w or LPS:3w, LPS:6w, and LPS:10w, respectively). Intranasal administrations were applied unilaterally to the left naris of each mouse, with the right side serving as an internal control. Another group of eight-week-old male mice underwent unilateral NC by brief cauterization and were housed for three, six, and 10 weeks (NC:3w, NC:6w, and NC:10w, respectively). For analyses of recovery, mice receiving LPS for 10 weeks were subsequently housed with no additional treatment for another two, six, and 10 weeks (LPS:10w+NT:2w, LPS:10w+NT:6w, and LPS:10w+NT:10w, respectively) or underwent NC (LPS: 10w+NC:10w).

### Immunostaining

Mice were anesthetized with ketamine (100 mg/kg body weight) and xylazine (10 mg/kg) and transcardially perfused with PBS, and then with 4% paraformaldehyde in PBS. Their heads were removed and placed in the same fixative at 4°C overnight. The rostral half of the calvaria and the nasal bone were then placed en block in 2× K-CX (Falma) for 2.5 h for decalcification and then washed with water for 6 h. The brains were cryoprotected with 30% sucrose in PBS (wt/vol) at room temperature overnight, embedded in OCT compound (Sakura Finetek USA Inc.), and maintained at –80°C until use.

Olfactory tissues were coronally cut on a cryostat into 20 μm slices, mounted on slide glasses, dried and stored at –80°C until use. The sections were rehydrated with TBST [10 mmol/l Tris-HCl (pH 7.4) and 100 mmol/l NaCl with 0.1% Tween 20], blocked with blocking buffer [1% bovine serum albumin for immunohistochemistry or 5% normal donkey serum (v/v) for immunofluorescence in TBST] at room temperature for 1 h, and incubated with primary antibodies diluted in blocking buffer overnight. The antibodies used in the present study are listed in Table 1. Sections were incubated with host-matched secondary antibodies (ImmPress rabbit-IgG, goat-IgG, or rat-IgG; Vector) at room temperature for 1 h and stained with ImmPACT SG (Vector). Nuclei were stained with nuclear fast red (Muto) for 1 min. Slices were cleared and coverslipped with HSR (Sysmex). Sections were examined with an EDC Eclipse light microscope equipped with a digital camera control unit, DS–Fi2/DS-L3 (Nikon).

**Table 1:** List of antibodies. Information of all antibodies used in this study is listed, including the name, host, dilution, source, and immunogen

Antibodyagainst	Host	Dilution	Source	Immunogen
F4/80	Rat	1:200	Abcam	Thioglycollate-stimulated peritoneal macrophages from C57/BL mice, clone A3-1
Ly-6G	Rat	1:200	AdipoGen	Purified mouse BALB/c neutrophils, clone Nimp-R14
IL-1β	Goat	1:200	R&D Systems	*E. coli*-derived recombinant mouse IL-1β/IL-1F2 Val118-Ser269
OMP	Goat	1:1000	Fujifilm Wako PureChemical Corp.	Rodent OMP
GAP43	Rabbit	1:1000	Novus Biologicals	C-terminal peptide of rat and mouse GAP43 with an N-terminalCys added to allow chemical coupling to KLH carrier protein
Calretinin	Rabbit	1:500	NeoMarkers	Recombinant full-length mouse calretinin protein
Iba-1	Rabbit	1:200	Wako	A synthetic peptide corresponding to the Iba-1 C-terminal sequence(PTGPPAKKAISELP)
GFAP	Rabbit	1:1000	Dako Agilent	GFAP isolated from cow spinal cord
TH	Rabbit	1:500	Millipore	Denatured TH from rat pheochromocytoma

For double immunostaining with fluorescence, Alexa Fluor 568-conjugated or 488-conjugated donkey anti-species IgGs (Thermo Fisher Scientific) were used as secondary antibodies (1:300). Nuclei were counterstained with 4′,6-diamidino-2-phenylindole (DAPI). The sections were coverslipped with fluorescence mounting medium (Dako Agilent) and imaged using a fluorescence microscope with structured illumination (BZ-X710; Keyence).

### Quantitative real-time RT-PCR assay

Mice from saline:10w, LPS:10w, and NC:10w groups were decapitated with a guillotine, and their brains were removed and dissected along the midline. OBs ipsilateral (left side) and contralateral (right side) to the treatment were obtained on ice under a stereomicroscope, snap frozen in liquid nitrogen, and stored at –80°C until use. Total RNA was isolated from the frozen OBs using a NucleoSpin RNA kit (Macherey-Nagel GmbH & Co KG) according to the manufacturer’s instructions. RNA quality was checked by examining absorbance values, and first-strand cDNA was synthesized from 1 μg total RNA with 200-U SuperScript III reverse transcriptase (Thermo Fisher Scientific) in the appropriate buffer in the presence of random primers, deoxyribonucleotide triphosphate mix, dithiothreitol, and 40-U RNase OUT (Thermo Fisher Scientific). Reverse transcription was performed at 25°C for 10 min, 50°C for 60 min, and 70°C for 15 min. Real-time PCR was performed using TB Green Premix Ex Taq II (Takara Bio Inc.), according to the manufacturer’s instructions, in a total volume of 25 μl with a Thermal Cycler Dice Real Time System II (Takara Bio Inc.) and 40 cycles of 95°C for 5 s and 60°C 30 s. The primer sequences are listed in Table 2. Transcript levels were normalized to those of *Gapdh* from corresponding samples. Data are expressed as relative fold change using the 2^-ΔΔ^*^CT^* calculation method.

**Table 2:** Primer sequences

Target	Direction	Sequence (5′ →3′)	Accession no.
IL-1β	Forward	CCTCACAAGCAGAGCACAA	NM_008361.4
	Reverse	CCAGCCCATACTTTAGGAAGAC	
TNFα	Forward	CTGAGTTCTGCAAAGGGAGAG	NM_001278601.1
	Reverse	CCTCAGGGAAGAATCTGGAAAG	
IL-10	Forward	TTGAATTCCCTGGGTGAGAAG	NM_010548.2
	Reverse	TCCACTGCCTTGCTCTTATTT	
Iba-1	Forward	GACGTTCAGCTACTCTGACTTT	NM_019467.3
	Reverse	GTTGGCCTCTTGTGTTCTTTG	
GFAP	Forward	GGAAGACACTGAAACAGGAGAG	NM_010277.3
	Reverse	AGAGCAGTCACAGGGTAAGA	
GAPDH	Forward	TCCTCAGTGTAGCCCAAGA	NM_001289726.1
	Reverse	GGAGAAACCTGCCAAGTATGA	

Information of all primers used in this study is listed, including the target, direction, sequence, and accession number.

### Image analyses and morphometry

All samples were randomly numbered so that the analyses were performed by an experimenter who was blind to sample identities.

#### Relative OB size

After cryoprotection, the nasal bones over the OBs were carefully removed under the microscope (Stemi305; Carl Zeiss AG) and the dorsal views of the ipsilateral and contralateral OBs were imaged with a digital camera (DS-Fi2/DS-L3; Nikon) attached to the microscope. Using these dorsal views, ipsilateral and contralateral OB areas were measured separately using Photoshop software (Adobe Systems Inc.). The number of pixels contained in the OB was measured and converted to area (mm^2^), and the ratio of the ipsilateral OB area to the contralateral area (ipsilateral OB area/contralateral OB area) was calculated. Values were compared among saline:3w, saline:6w, and saline:10w (*n* = 4, 3, and 4, respectively), LPS:3w, LPS:6w, and LPS:10w (*n* = 4, 3, and 4, respectively); and NC:3w, NC:6w, and NC:10w (*n* = 4, 4, and 5, respectively), and among LPS:10w, LPS:10w+NT:10w, and LPS:10w+NC:10w (*n* = 4) or LPS:10w, LPS:10w+NT:2w, LPS:10w+NT:6w, and LPS:10w+NT:10w (*n* = 4).

#### OB layer areas

Nuclei in coronal sections of the OBs were stained with nuclear fast red. The number of pixels enclosed by the boundaries of each layer was measured and converted to area (mm^2^) using Photoshop software (Adobe Systems Inc.). As the rostral parts of the OB are much smaller and caudal parts have much thinner ONL, layer analyses were performed using the sections from the “middle OBs” that included 3- to 4-mm^2^ area of the whole OB and ≥0.5 mm^2^ area of the ONL on the contralateral side. Three these sections per mouse were selected for the statistical analysis. The ratio of each layer was determined by dividing the area from the ipsilateral side by the area from the contralateral side. Values were compared among saline:10w, LPS:10w, and NC:10w, or LPS:10w, LPS:10w+NT:10w, and LPS:10w+NC:10w (*n* = 3).

#### sEPL area

Sections from the middle OBs were stained with DAPI and for calretinin to label mitral cells, granule cells with synaptic connections with mitral cells, and some periglomerular cells. Within the EPL, the calretinin-positive area was regarded as the deep EPL (dEPL) and the calretinin-negative area was regarded as the sEPL (Malz et al., 2000). The pixel numbers enclosed by the boundaries of sEPL were measured and converted to the area (mm^2^) by using Photoshop software (Adobe Systems Inc.). The ratio of the sEPL was determined by dividing the area of the sEPL by the total EPL area. Values were compared among saline:10w, LPS:10w, and NC:10w or LPS:10w, LPS:10w+NT:10w, and LPS:10w+NC:10w (*n* = 3).

#### Olfactory marker protein (OMP)-positive length

Coronal sections of the OE were stained with DAPI and for the OMP expressed by mature OSNs. Two OE sections per each mouse that were 300–600 μm rostral to the anterior tip of the OB were selected for the analysis. The length along the surface of the OE including the septal OE, the first, second and third turbinates (total OE), and the length along the OMP-positive epithelium within the total OE were measured using ImageJ software. The ratio of OMP-positive length was determined by dividing the length of the OMP-positive epithelium by the total OE length. Values were compared among LPS:10w, LPS:10w+NT:10w, and LPS:10w+NC:10w (*n* = 3).

### Experimental design and statistical analysis

Comparisons of the relative OB sizes among saline, LPS, and NC treatments were statistically analyzed by two–way analysis of variance (two main effects of treatment and time), followed by Tukey’s HSD *post hoc* tests for multiple comparisons. A *p* < 0.05 indicated a significant difference. The areas of each layer of the OB, ratios of sEPLs, transcript amounts, time courses of the changes in the relative OB size, and ratios of OMP-positive length to total OE length were statistically analyzed using Tukey’s HSD tests. Statistical analyses were performed using Statistica software (Dell Software). Values are reported as means ± SEMs.

## Results

### OE inflammation and loss of OSNs

**Figure 1.**
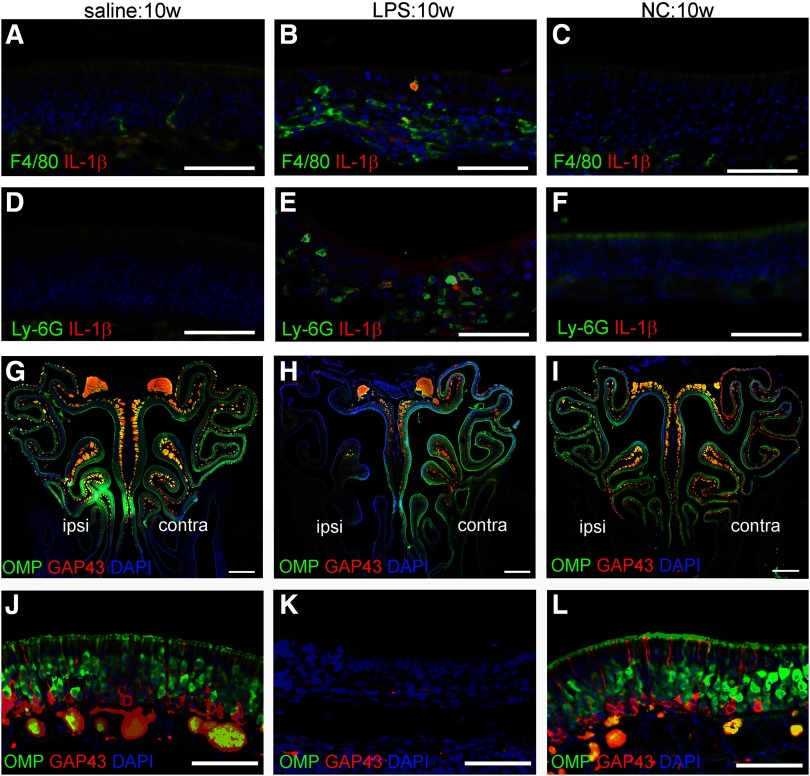
Nasal inflammation and loss of OSNs. ***A*–*C***, Immunofluorescence for F4/80 (green), IL-1β (red), and nuclei (DAPI, blue) in the ipsilateral OE in saline:10w (***A***), LPS:10w (***B***) and NC:10w (***C***). F4/80-immunopositive macrophages infiltrated the OE in LPS:10w (***B***) but not in saline:10w (***A***) or NC:10w (***C***). Some F4/80-immunopositive macrophages in the OE expressed IL-1β in LPS:10w (***B***). ***D*–*F***, Immunofluorescence for Ly-6G (green), IL-1β (red), and nuclei (DAPI, blue) in the ipsilateral OE in saline:10w (***D***), LPS:10w (***E***), and NC:10w (***F***). Ly-6G-immunopositive neutrophils infiltrated the OE in LPS:10w (***E***) but not in saline:10w (***D***) or NC:10w (***F***). A few Ly-6G-immuopositive neutrophils in the OE expressed IL-1β in LPS:10w (***E***). ***G*–*I***, Coronal sections of OE stained for OMP (green), GAP43 (red), and nuclei (DAPI; blue) in saline:10w (***G***), LPS:10w (***H***), and NC:10w (***I***). OMP- and GAP43-immunopositive mature and immature OSNs were lost in the ipsilateral OE in LPS:10w (***H***) but not in saline:10w (***G***) or NC:10w (***I***). ***J*–*L***, Magnified views of ipsilateral OE stained for OMP (green), GAP43 (red), and nuclei (DAPI, blue). Scale bars: 50 μm (***A*–*F***, ***J*–*L***) and 500 μm (***G*–*I***).

Macrophages (F4/80 positive) and neutrophils (Ly-6G positive) did not infiltrate the OE in animals administered saline (Fig. 1*A*,*D*) but accumulated in the OE and lamina propria, some of which expressed interleukin-1β (IL-1β), of LPS:10w (Fig. 1*B*,*E*). Few if any macrophages or neutrophils were observed in OE of NC:10w (Fig. 1*C*,*F*), indicating that NC did not induce nasal inflammation.

In the saline:10w, mature (OMP positive) and immature [growth-associated protein 43 (GAP43) positive] OSNs were detected in the OE (Fig. 1*G*,*J*), whereas these were lost from the ipsilateral OE in LPS:10w (Fig. 1*H*,*K*), consistent with our previous study. OSNs were detected in the ipsilateral and contralateral OE in NC:10w (Fig. 1*I*,*L*), indicating that 10 weeks of NC did not induce a loss of OSNs.

### Gross atrophy of the OB

**Figure 2.**
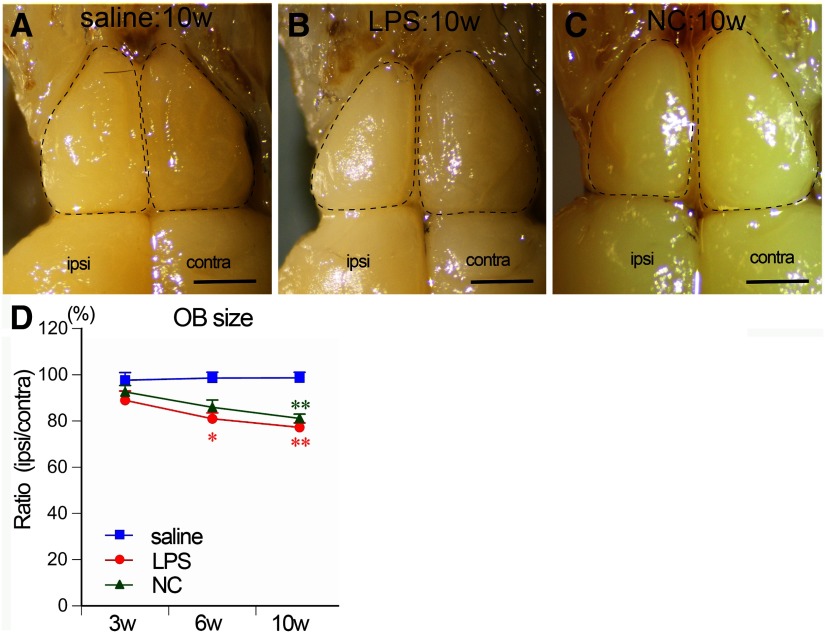
Atrophy of the OB. ***A*–*C***, Dorsal views of the OBs of saline:10w (***A***), LPS:10w (***B***), and NC:10w (***C***). Ipsilateral OBs atrophied in LPS:10w (***B***) and NC:10w (***C***). Scale bars: 1 mm. ***D***, Graph presents the time course of changes in the OB size. Ratios of ipsilateral OB areas to the contralateral ones gradually decreased in LPS:10w and NC:10w; *p < 0.05, **p < 0.01 versus saline:10w.

In saline-treated mice, ipsilateral and contralateral OBs were similar in size, because the ipsilateral areas were almost 100% relative to the contralateral OB areas at all time points examined (97.6 ± 3.3%, 98.6 ± 2.5%, and 98.7 ± 2.4% of the contralateral values in saline:3w, saline:6w, and saline:10w, respectively; Fig. 2*A*,*D*), indicating that the administration procedure did not induce atrophy. In LPS-treated mice, the ipsilateral OB areas gradually decreased to 81.0 ± 2.5% and 77.2 ± 1.9% of the contralateral values at six and 10 weeks, respectively, which were significantly lower than in saline-treated mice at the same time points (Fig. 2*B*,*D*). In NC mice, the areas of ipsilateral OB significantly decreased to 81.1 ± 1.8% of the contralateral values at 10 weeks (Fig. 2*C*,*D*). The contralateral OB sizes did not significantly differ among saline-treated, LPS-treated, and NC mice (data not shown). These results indicate that LPS and NC treatments similarly induced atrophy of the ipsilateral OB within 10 weeks.

### Specific layer shrinkage

**Figure 3.**
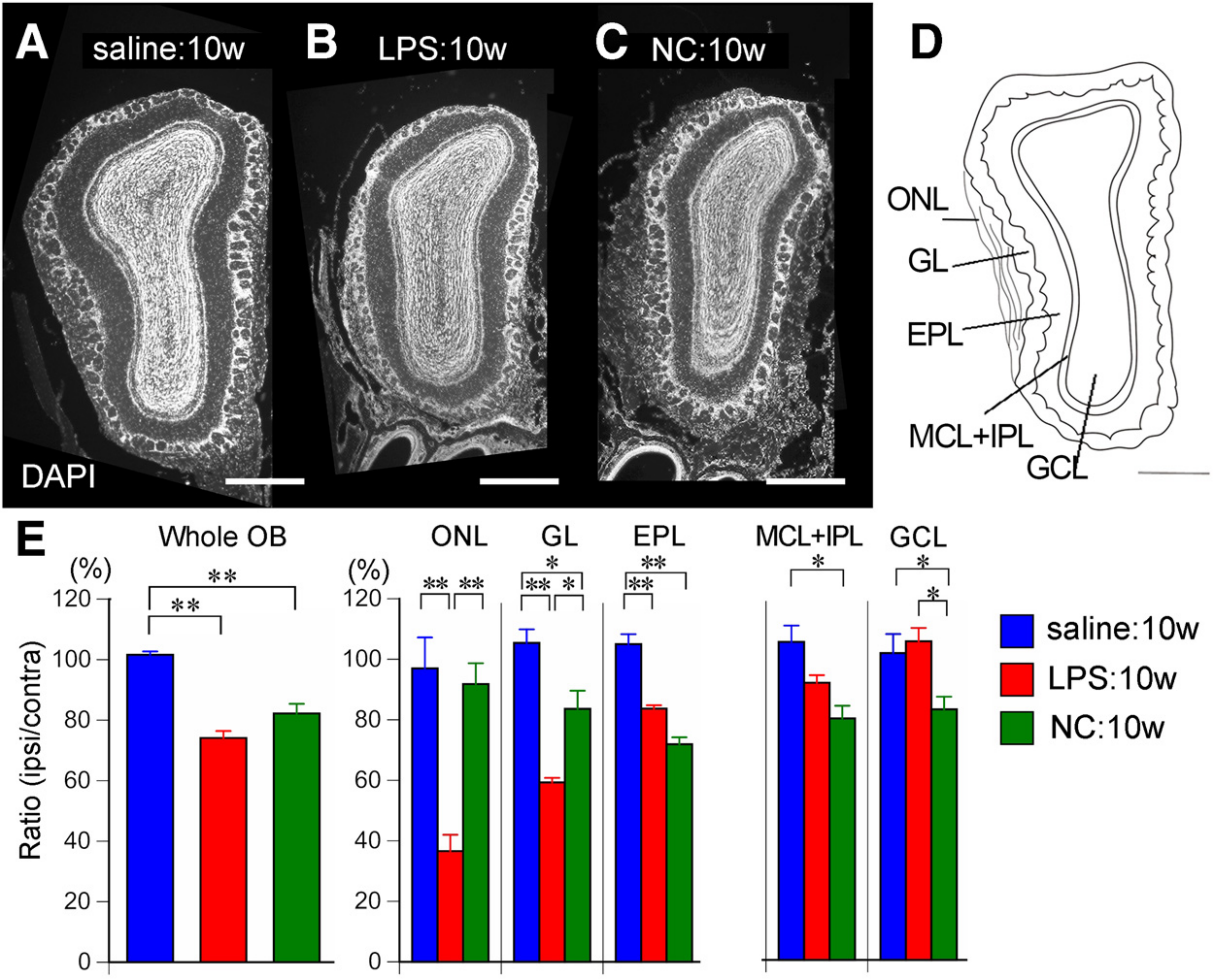
Shrinkage of layers in the OB. ***A*–*C***, Coronal sections of the ipsilateral OBs stained with DAPI. ***D***, Schematic image of the OB representing each layer. ***E***, Graphs present the ratios of the ipsilateral layer areas to the contralateral ones. Whole OB contains all layers. ONL, GL, and EPL shrank in the LPS:10w, whereas GL, EPL, MCL+IPL, and GCL shrank in the NC:10w; *p < 0.05, **p < 0.01. Scale bars: 500 μm (***A*–*D***).

To examine which layers of the OB contributed to the observed atrophy, we measured the area of each layer using coronal sections from the middle of the ipsilateral and contralateral OBs (Fig. 3*A–D*). These measurements confirmed that saline treatment did not reduce the area of the whole OB [sum of ONL, GL, EPL, mitral cell layer (MCL) plus the internal plexiform layer (IPL; MCL+IPL) and GCL; 101.6 ± 1.1% of contralateral values], whereas ipsilateral areas were significantly lower both in LPS:10w (74.1 ± 2.3%) and NC:10w (82.2 ± 3.2%; Fig. 3*E*). In LPS:10w, the areas of the ipsilateral ONL, GL, and EPL relative to their contralateral counterparts (36.6 ± 5.4%, 59.3 ± 1.5%, and 83.7 ± 1.1%, respectively) were significantly lower than in the saline:10w (97.0 ± 10.2%, 105.4 ± 4.5%, and 105.0 ± 3.3%, respectively; Fig. 3*E*). The ratios for MCL+IPL and GCL did not significantly change in the LPS:10w compared with those in the saline-treated controls. Whereas NC did not alter the ratios for ONL, those for GL, EPL, MCL+IPL, and GCL (83.6 ± 6.0%, 71.9 ± 2.3%, 79.8 ± 2.5%, and 82.8 ± 4.2%, respectively) were significantly lower than in saline-treated controls (105.4 ± 4.5%, 105.0 ± 3.3%, 105.1 ± 5.4%, and 101.4 ± 6.3%, respectively; Fig. 3*E*). Thus, different layers contributed to the OB atrophy observed in LPS-treated and NC mice: LPS induced shrinkage of ONL, GL, and EPL, and NC induced shrinkage of GL, EPL, MCL+IPL, and GCL (Fig. 3*E*).

**Figure 4.**
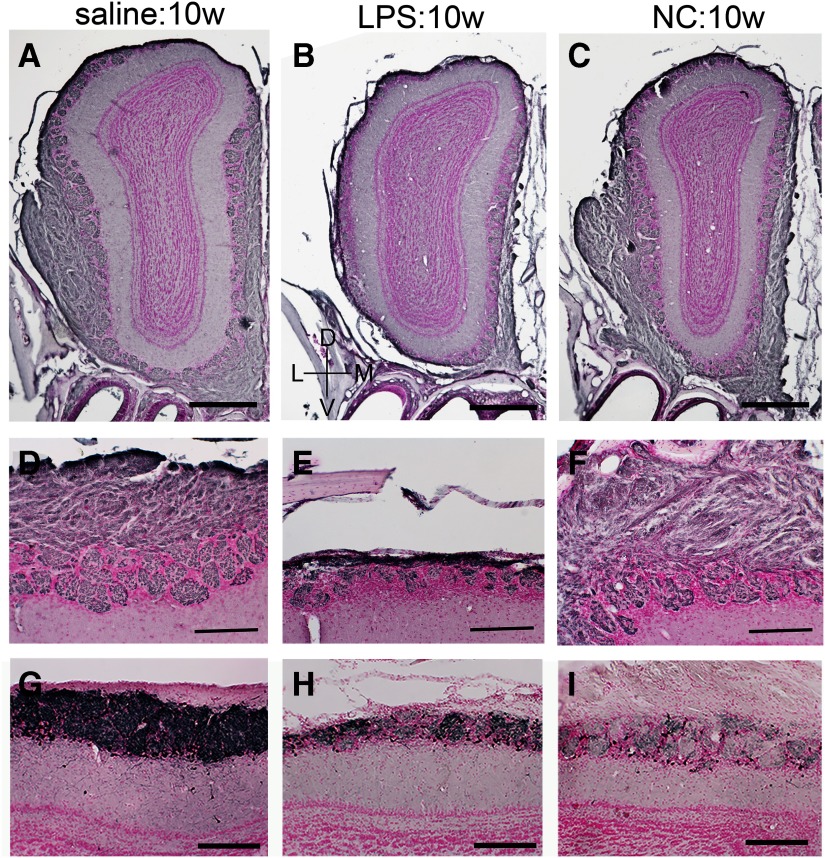
OMP and TH expression in the OB. ***A*–*F***, Immunohistochemistry for OMP, representing ONL and GL, in ipsilateral OBs from saline:10w (***A***, ***D***), LPS:10w (***B***, ***E***), and NC:10w (***C***, ***F***). OMP-immunopositive axon terminals of OSNs were lost particularly in the lateral side of the ipsilateral OB in LPS:10w (***B***, ***E***) but not in saline:10w (***A***, ***D***) or NC:10w (***C***, ***F***). ***D*–*F***, Magnified views of lateral side of the OBs in ***A*–*C***. ***G*–*I***, Immunohistochemistry for TH expressed by some juxtaglomerular cells. TH expression decreased in LPS:10w (***H***) and in NC:10w (***I***) compared with that in the saline:10w (***G***). Nuclei were stained with nuclear fast red. Scale bars: 500 μm (***A*–*C***) and 200 μm (***D*–*I***).

We confirmed the shrinkage of ONL and GL by immunohistochemistry for OMP, a marker for mature OSNs. The ONL and GL were immunopositive for OMP in saline-treated controls (Fig. 4*A*,*D*), whereas the staining in the lateral ONL of the ipsilateral OB was almost completely lost in LPS:10w (Fig. 4*B*,*E*). The number and the size of glomeruli were remarkably decreased in the lateral side of the ipsilateral OB, whereas the medial side was largely intact in LPS:10w. Immunoreactivity in ONL in the NC:10w (Fig. 4*C*,*F*) was similar to that in the saline:10w, indicating that the OSNs were histologically intact in NC:10w.

A subpopulation of juxtaglomerular cells that extend their dendrites into glomeruli expresses tyrosine hydroxylase (TH) in an activity-dependent manner (Cho et al., 1996), such that TH immunostaining is detected in the GL in saline-treated controls (Fig. 4*G*). TH expression in the GL was weaker in LPS:10w than in saline:10w, but was much weaker in NC:10w (Fig. 4*H*,*I*).

### Thinning of the sEPL

**Figure 5.**
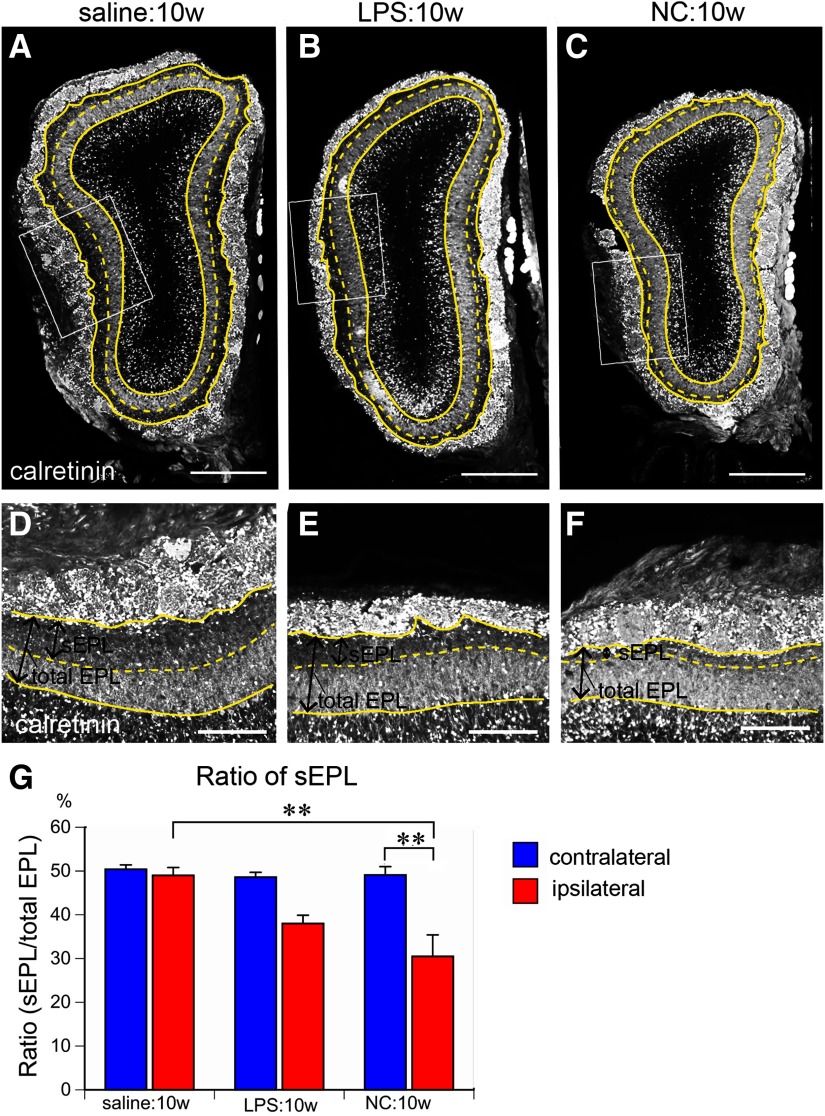
Shrinkage of sEPL. ***A*–*F***, Coronal sections of the ipsilateral OB stained for calretinin (expressed by mitral cells and some granule cells connecting to mitral cells in the dEPL). Solid yellow lines represent the borders of the GL and EPL or EPL and MCL. Dotted yellow lines represent the borders of sEPL and dEPL. ***D*–*F***, Magnified views of areas indicated by rectangles in ***A*–*C***. The sEPL preferentially shrank in LPS:10w and NC:10w. ***G***, Graph presents the ratios of sEPL to total EPL. The ratios of sEPL to total EPL decreased in ipsilateral EPLs in LPS:10w and NC:10w; **p < 0.01. Scale bars: 500 μm (***A*–*C***) and 200 μm (***D*–*F***).

We further characterized the shrinkage of the EPL after 10 weeks of LPS and NC to determine which sublayer was more vulnerable to nasal inflammation and odor deprivation. The sEPL can be distinguished from the dEPL by its weaker calretinin immunostaining, which is expressed by mitral cells and connecting granule cells (Fig. 5*A–F*; Malz et al., 2000). In saline-treated controls, the sEPL was almost 50% of the total EPL in ipsilateral and contralateral OBs (Fig. 5*A*,*D*,*G*). By contrast, this was reduced to 38.0% (Fig. 5*B*,*E*,*G*) and 30.5% (Fig. 5*C*,*F*,*G*) in the ipsilateral OBs after 10 weeks of LPS and NC, respectively. Thus, the superficial layer of the EPL is affected by inflammation and odor deprivation, with greater sensitivity to odor deprivation.

### Glial responses and cytokine expression

**Figure 6.**
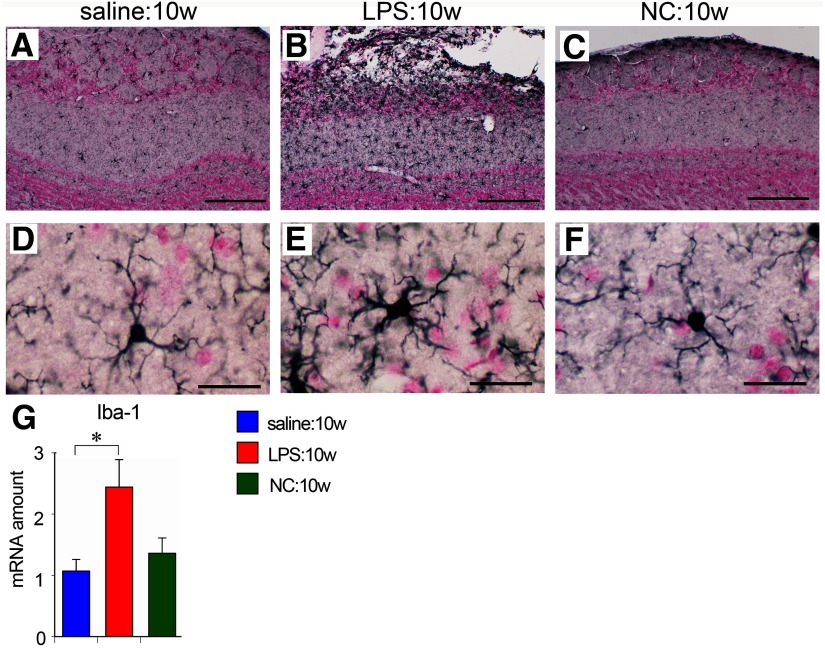
Microglial activation in the OB. ***A*–*F***, Immunohistochemistry for Iba-1 in ipsilateral OBs. Microglia were activated in LPS:10w (***B***, ***E***) but not in NC:10w (***C***, ***F***) compared with those in the saline:10w (***A***, ***D***). Representative microglia are magnified in ***D*–*F***. Nuclei were stained for nuclear fast red. Scale bars: 200 μm (***A*–*C***) and 20 μm (***D*–*F***). ***G***, Graph presents the relative amounts of transcript for Iba-1 in ipsilateral OBs. The Iba-1 transcript amount in the LPS:10w was ∼2.5 times than in the saline:10w; *p < 0.05.

Microglia have a small cell body and thin radially projecting processes, and are distributed evenly throughout the brain and OB under normal conditions, as seen in saline-treated control (Fig. 6*A*,*D*). In LPS:10w, microglia were activated with larger cell bodies and thicker processes, particularly on the lateral side of the ipsilateral OB (Fig. 6*B*,*E*). Quantitative PCR analysis revealed that the amount of transcript for ionized calcium-binding adaptor molecule-1 (Iba-1) was ∼2.5 times higher in the ipsilateral OBs of LPS:10w than in the saline controls (Fig. 6*G*). By contrast, microglia appeared normal, and the Iba-1 transcript amount did not change in the NC:10w (Fig. 6*C*,*F*,*G*).

**Figure 7.**
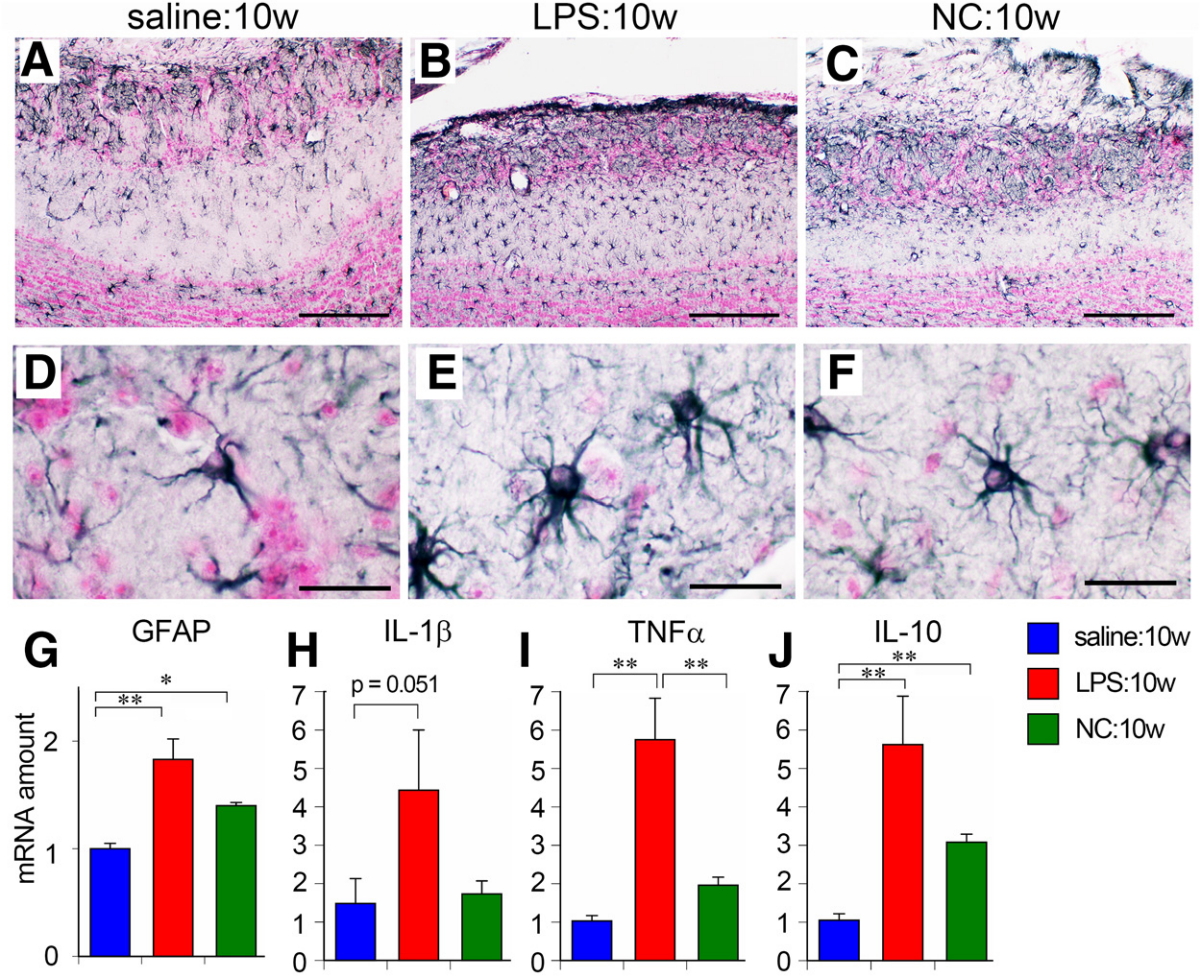
Astrocytic hypertrophy and neuroinflammation in the OB. ***A*–*F***, Immunohistochemistry for GFAP in ipsilateral OBs. Astrocytes were hypertrophic in LPS:10w (***B***, ***E***) but not prominently in NC:10w (***C***, ***F***) compared with those in the saline:10w (***A***, ***D***). Representative astrocytes are magnified in ***D*–*F***. Nuclei were stained for nuclear fast red. Scale bars: 200 μm (***A*–*C***) and 20 μm (***D*–*F***). ***G–J***, Graphs present the relative amounts of transcript for GFAP, IL-1β, TNFα, and IL-10 in ipsilateral OBs. The transcript amounts of these molecules were significantly higher in the LPS:10w than in the saline:10w. The transcript amount of IL-10 was also higher in NC:10w than in saline:10w; *p < 0.05, **p < 0.01.

Astrocytes were located mainly in the GL, sEPL and GCL of saline-treated (Fig. 7*A*,*D*) and NC-treated mice (Fig. 7*C*,*F*). However, hypertrophic astrocytes were distributed throughout the EPL as well as the GL in LPS:10w (Fig. 7*B*,*E*). Quantitative PCR analysis revealed that the amounts of transcript for glial fibrillary acidic protein (GFAP) significantly increased in LPS:10w and NC:10w (Fig. 7*G*), suggesting that astrocytes reacted to NC, although their morphology was not prominently altered.

As we observed robust glial activation in the ipsilateral OBs of LPS:10w, we expected to observe another sign of neuroinflammation. We performed quantitative PCR to measure the expression of IL-1β, TNFα, IL-6, and COX-2 as representative proinflammatory factors and of IL-10, TGFβ, and BDNF as representative anti-inflammatory factors. In the ipsilateral OBs, 4.4 times more IL-1β and 5.8 times more TNFα were expressed in the LPS:10w than in the saline controls (*p* = 0.051 for IL-1β and *p* < 0.01 for TNFα), with no change detected in the NC:10w (Fig. 7*H*,*I*). We also found that IL-10 expression was six and three times higher in the LPS:10w and NC:10w, respectively (Fig. 7*J*). There were no changes in the expression levels of other inflammatory factors examined with LPS or NC treatment. These results indicate that 10 weeks of LPS administration, but not NC, induces neuroinflammation in the OB.

### Remission of nasal inflammation and neuroinflammation

The OB recovers from LPS-induced atrophy once nasal inflammation subsides (Hasegawa-Ishii et al., 2019). In the present study, we examined whether odor deprivation is related to OB recovery.

**Figure 8.**
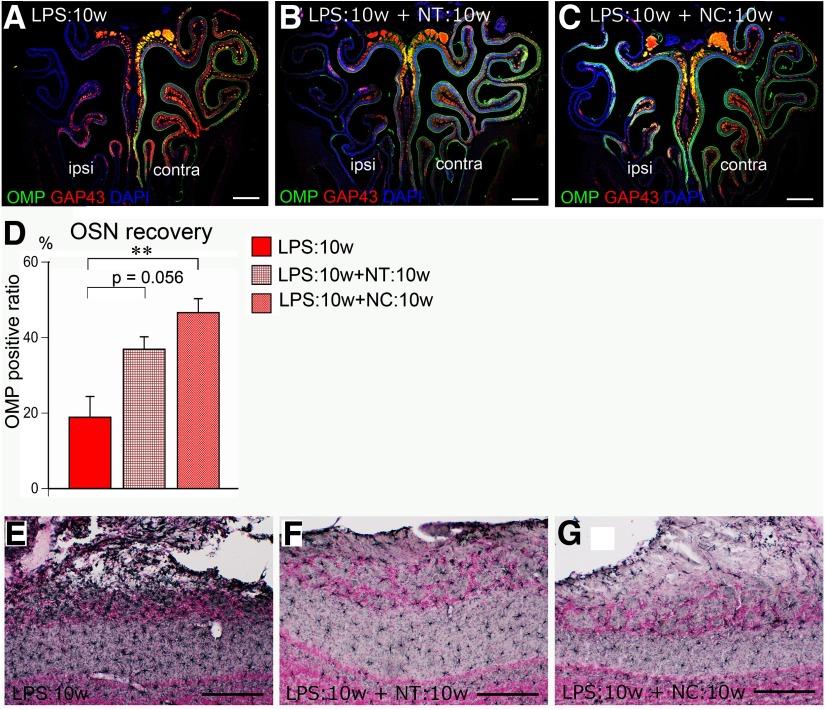
Remission of nasal-inflammation and neuro-inflammation. ***A*–*C***, Coronal sections of the OE stained for OMP (green), GAP43 (red), and nuclei (DAPI, blue). OMP- and GAP43-immunopositive mature and immature OSNs are lost in LPS:10w (***A***) and regenerated in a patchy manner in the presence (***B***) and absence (***C***) of odor input. ***D***, Graph presents the OMP-immunopositive length relative to the total length of ipsilateral OE. The ratio for the OMP-immunopositive length increased in the presence (LPS:10w+NT:10w) or absence (LPS:10w+NC:10w) of odor input; **p < 0.01. ***E*–*G***, Immunohistochemistry for Iba-1 in the ipsilateral OB. Microglia were activated in LPS:10w (***E***) but returned to normal in LPS:10w+NT:10w (***F***) and in LPS:10w+NC:10w (***G***). Nuclei were stained with nuclear fast red. Scale bars: 500 μm (***A*–*C***) and 200 μm (***E*–*G***). In the OEs of NC:3w and NC:10w, there are more calretinin-immunopositive OSNs on the closed side than on the open side. Similarly, there are more calretinin-immunopositive OSNs in the OE of LPS:10w+NC:10w than in LPS:10w+NT:10w (Extended Data Fig. 8-1).

First, we examined whether nasal inflammation subsides in the presence (LPS:10w+NT:10w) or absence (LPS:10w+NC:10w) of odor input. Very few F4/80-positive macrophages or Ly-6G-positive neutrophils were detected in the OE in the LPS:10w+NT:10w and LPS:10w+NC:10w (data not shown), indicating that nasal inflammation subsided regardless of odor input. To examine the recovery of OSNs, we calculated the OMP-positive length relative to the entire OE length. In LPS:10w, 18.9 ± 5.5% of OE length was OMP positive (Fig. 8*A*,*D*), which increased to 36.9 ± 3.3% in the LPS:10w+NT:10w and significantly increased to 46.6 ± 3.7% in the LPS:10w+NC:10w (Fig. 8*B–D*). These results indicate that OSNs were able to recover from inflammation-induced damage in the presence and absence of odor input.

10.1523/ENEURO.0403-19.2020.f8-1 Extended Data Figure 8-1 Increased number of calretinin-positive OSNs in the closed side of NC. ***A*–*D***, Coronal sections of the OE stained for OMP (green), calretinin (red), and nuclei (DAPI, blue). Calretinin-immunopositive intermediate OSNs increased in the closed side of NC:3w and NC:10w compared with that in the open side. Note that the number of OMP- and calretinin-double immunopositive cells increase in the closed side of NC mice. ***E*–*G***, Coronal sections of the OE stained for calretinin (red) and nuclei (DAPI, blue). Calretinin-immunopositive OSNs are lost in LPS:10w (***E***) and regenerated in a patchy manner in the presence (***F***) and absence (***G***) of odor input. Note that calretinin-immunopositive OSNs remarkably increased in number in LPS:10w+NC:10w. Download Figure 8-1, TIF file.

We also examined neuroinflammation in the OB during this recovery period by assessing glial activation. Compared with that in the LPS:10w, the size, numbers, and morphology of microglia returned to normal in both the LPS:10w+NT:10w and LPS:10w+NC:10w (Fig. 8*E–G*). Similarly, the hypertrophy and accumulation of astrocytes in the EPL in the LPS:10w were reversed in the LPS:10w+NT:10w and LPS:10w+NC:10w (data not shown). These results indicate that neuroinflammation subsided in the OB in the presence or absence of odor input.

### Recovery from OB atrophy

**Figure 9.**
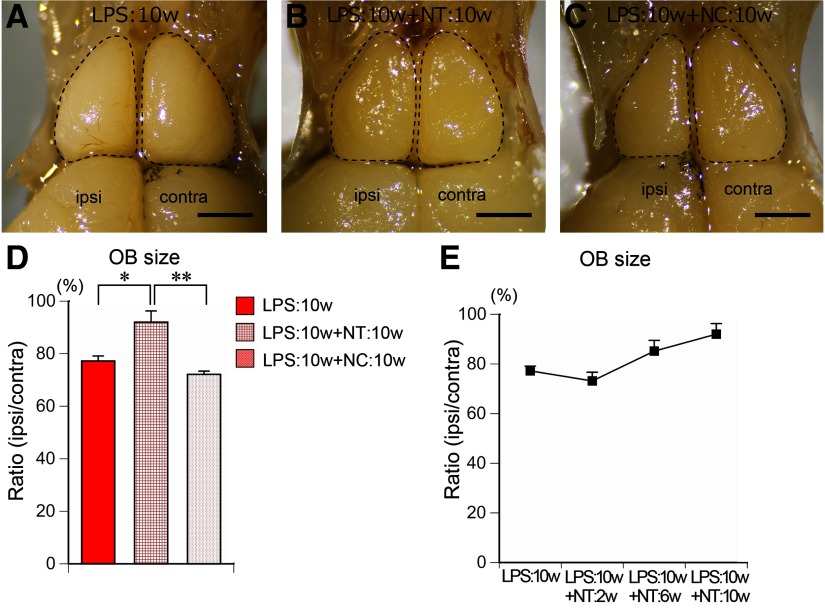
Recovery of OB in the presence or absence of odor input. ***A*–*C***, Dorsal views of the OBs from LPS:10w (***A***), LPS:10w+NT:10w (***B***), and LPS:10w+NC:10w (***C***). Ipsilateral OB recovered from atrophy in the presence (***B***) but not in the absence (***C***) of odor input. Scale bars: 1 mm. ***D***, Graph presents the ratios of the ipsilateral OB areas to the contralateral ones. The ratio significantly increased in the presence (LPS:10w+NT:10w) but not in the absence (LPS:10w+NC:10w) of odor input; *p < 0.05, **p < 0.01. ***E***, Graph presents the time course of the ratio of the ipsilateral OB area to the contralateral one after 10 weeks of LPS administration.

We next examined whether the atrophy of the OB recovers in the presence or absence of odor input during the recovery phase. The average size of the ipsilateral OB significantly increased from 77.2 ± 1.9% of the contralateral side in LPS:10w to 92.0 ± 4.3% in the LPS:10w+NT:10w (Fig. 9A,*B*,*D*). The areas of OB gradually increased during 10 weeks of nontreatment (Fig. 9*E*). By contrast, the OB size did not recover in the absence of odor input, because the average ipsilateral OB was 72.1 ± 1.2% of the contralateral size in the LPS:10w+NC:10w (Fig. 9*C*,*D*).

**Figure 10.**
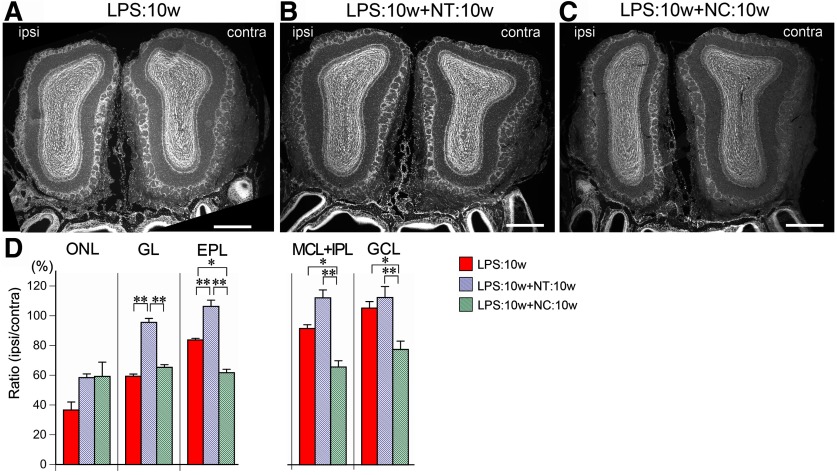
Recovery of each layer in the presence or absence of odor input. ***A*–*C***, Coronal sections of the OBs stained with DAPI from LPS:10w (***A***), LPS:10w+NT:10w (***B***), and LPS:10w+NC:10w (***C***). Ipsilateral OBs recovered from atrophy in the presence (***B***) but not in the absence (***C***) of odor input. Ipsilateral OBs further shrank in the absence of odor input (***C***). Scale bars: 500 μm. ***D***, Graphs present the ratios of the ipsilateral layer areas to the contralateral ones. ONL areas did not change among LPS:10w, LPS:10w+NT:10w, and LPS:10w+NC:10w, while GL and EPL, which shrank in LPS:10w, recovered in the presence (LPS:10w+NT:10w) but not in the absence (LPS:10w+NC:10w) of odor input. EPL area further shrank in the absence of odor input (LPS:10w+NC:10w). MCL+IPL and GCL, which did not shrink in LPS:10w, shrank in the absence (LPS:10w+NC:10w) but not in the presence (LPS:10w+NT:10w) of odor input; *p < 0.05, **p < 0.01.

To determine whether the recovery was layer specific, we compared the areas of each layer in coronal sections of the OBs among the LPS:10w, LPS:10w+NT:10w, and LPS:10w+NC:10w (Fig. 10*A–C*). The ONL was slightly but not significantly thicker in the LPS:10w+NT:10w and LPS:10w+NC:10w, at 58.4 ± 2.5%, and 59.2 ± 9.6% of the contralateral layers, respectively (Fig. 10*D*). However, the areas of the GL and EPL significantly increased to 95.5 ± 2.7% and 106.3 ± 4.2%, respectively, in the LPS:10w+NT:10w, as expected on the basis of a previous study. By contrast, GL was not different in the LPS:10w+NC:10w compared with that in the LPS:10w (Fig. 10*D*), whereas the EPL further decreased to 61.7 ± 2.3% of that of the contralateral side (Fig. 10*D*). Moreover, the MCL+IPL and GCL, which were not decreased in the LPS:10w, decreased in the LPS:10w+NC:10w compared with those in the LPS:10w and LPS:10w+NT:10w (Fig. 10*D*), suggesting that LPS:10w+NC:10w mice suffered from tandem insults of LPS and NC. These results indicate that the ONL recovered in the presence and absence of odor input, but the GL and EPL needed odor input for recovery.

**Figure 11.**
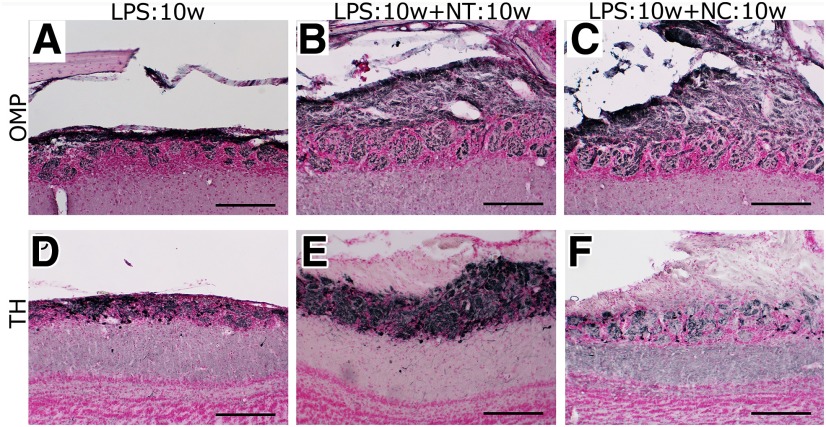
Recovery of OMP and TH expression in the OB. ***A*–*C***, Immunohistochemistry for OMP in ipsilateral OB from LPS:10w (***A***), LPS:10w+NT:10w (***B***), and LPS:10w+NC:10w (***C***). OMP expression recovered in LPS:10w+NT:10w (***B***) and LPS:10w+NC:10w (***C***) compared with that in the LPS:10w (***A***). ***D*–*F***, Immunohistochemistry for TH expressed by some juxtaglomerular cells. TH expression recovered in LPS:10w+NT:10w (***E***) but further decreased in LPS:10w+NC:10w (***F***) compared with that in the LPS:10w (***D***). Nuclei were stained with nuclear fast red. Scale bars: 200 μm.

The recovery of the ONL and GL in the LPS:10w+ NT:10w and LPS:10w+NC:10w was confirmed by immunohistochemistry for OMP (Fig. 11*A–C*). In addition, TH expression was restored in the LPS:10w+NT:10w, but not in the LPS:10w+NC:10w (Fig. 11*D–F*).

**Figure 12.**
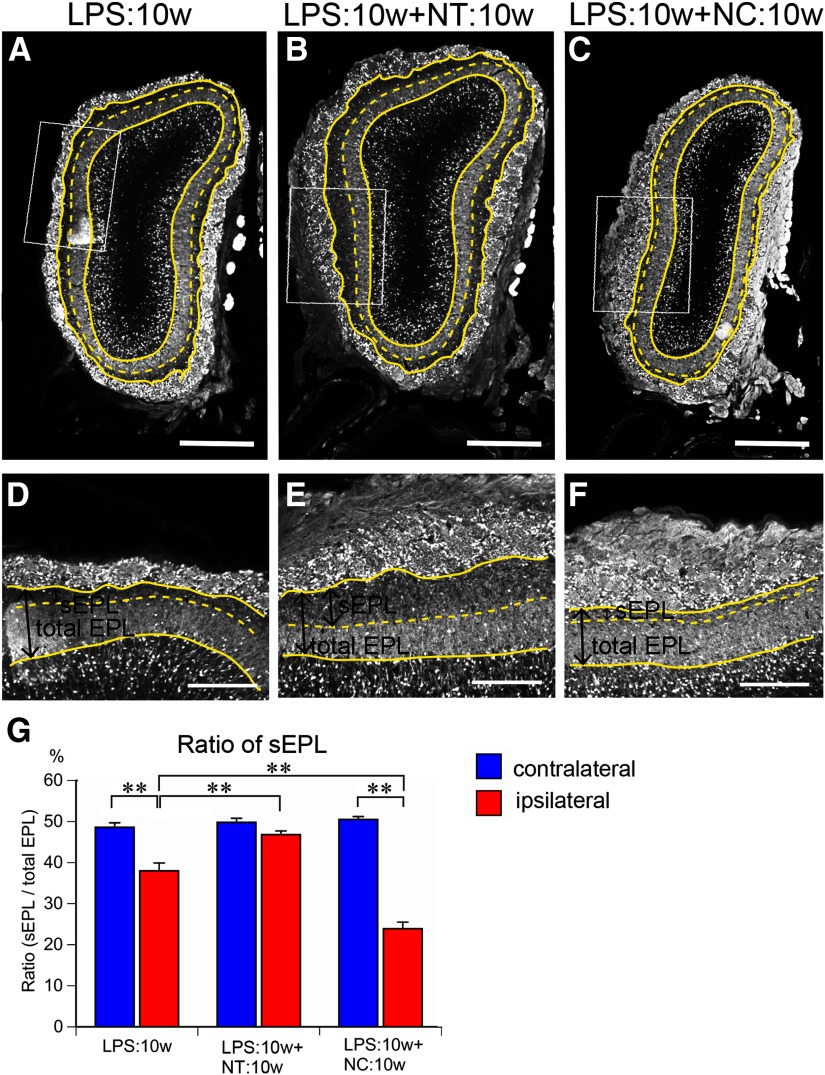
Recovery of sEPL in the presence or absence of odor input. ***A*–*F***, Coronal sections of ipsilateral OBs stained for calretinin. Solid yellow lines represent the borders of GL and EPL or EPL and MCL. Dotted yellow lines represent the borders of sEPL and dEPL. ***D*–*F***, Magnified views of EPL indicated by rectangles in ***A*–*C***. The sEPL area shrank in LPS:10w (***A***) and recovered in the presence (***B***) but not in the absence (***C***) of odor input. Scale bars: 500 μm (***A*–*C***) and 200 μm (***D*–*F***). ***G***, Graph presents the ratios of sEPL to total EPL. The ratios of sEPL to total EPL significantly decreased in ipsilateral EPL in LPS:10w and LPS:10w+NC:10w, but not in the LPS:10w+NT:10w; **p < 0.01.

The reduced area of the sEPL in the ipsilateral OBs in the LPS:10w (Figs. 5*B*,*E*,*G*, 12*A*,*D*,*G*) recovered to 46.8% of the total EPL in the LPS:10w+NT:10w, which was similar to that of the contralateral OB (Fig. 12*B*,*E*,*G*). By contrast, the area of the sEPL further decreased to 23.9% of the total EPL in the ipsilateral OBs of the LPS:10w+ NC:10w, whereas the ratio did not change in the contralateral OBs (Fig. 12*C*,*F*,*G*).

## Discussion

Chronic nasal inflammation induces a loss of OSNs and results in neuroinflammation and gross atrophy of the OB (Hasegawa-Ishii et al., 2019). The results from the present study extended these findings and demonstrated that chronic nasal inflammation and long–term odor deprivation have differential effects. LPS-induced chronic nasal inflammation caused shrinkage of superficial OB layers (ONL, GL, and EPL) contributing to OB atrophy, whereas NC-induced long-term odor deprivation caused shrinkage of all layers except ONL (GL, EPL, MCL+IPL, and GCL), resulting in atrophy without inflammation or OSN loss.

The shrinkage of the ONL and GL in LPS-treated mice was attributed to the retraction of the axons of OSNs. The shrinkage of the GL was also due to the retraction of dendrites of OB projection neurons in LPS-treated and NC mice. By contrast, the shrinkage of the GCL after 10 weeks of odor deprivation may have resulted from reduced neurogenesis of OB interneurons and/or survival of newly generated granule cells (Yamaguchi and Mori, 2005), processes that are facilitated by odor enrichment (Woo et al., 2006). Although the ONL, GL, and GCL were differentially affected, both LPS and NC treatments induced shrinkage of the EPL, particularly the sEPL. The superficial one-third of the EPL receives projections from tufted cells, whereas the deeper layers comprise dendrodendritic synapses from mitral cells. Thus, neuronal circuits involving tufted cells are preferentially affected by both chronic nasal inflammation and long-term odor deprivation.

The mechanism for sEPL shrinkage likely involved a loss of OSN activity. OSN activity is eliminated by odor deprivation via NC, while it may not be completely eliminated as a result of inflammation in LPS-treated mice. This idea is supported by the results showing that NC had a much greater effect on the sEPL area than LPS treatment, and induced further shrinkage of MCL+IPL and GCL. NC also reduced TH expression in the GL to a greater extent than LPS treatment, indicating that the odor-induced activity of juxtaglomerular cells was much lower in the NC mice than in the LPS-treated mice.

The results from a previous study suggested that glial activation and proinflammatory cytokines, a hallmark of neuroinflammation, contribute to EPL shrinkage (Hasegawa-Ishii et al., 2019). However, the findings from the present study failed to support this and further demonstrated that this does not occur with odor deprivation. Moreover, the EPL did not recover but rather underwent further shrinkage in the absence of odor input despite the alleviation of inflammation after the cessation of LPS administration. These data suggest that a reduction in glial activation does not contribute to recovery in the OB. By contrast, activated glial cells may support tissue homeostasis in the OB. In the present study, IL-10, an anti-inflammatory cytokine, was upregulated in LPS:10w and NC:10w. Given that the tufted cells were vulnerable to nasal inflammation and odor deprivation, IL-10 may be produced by some cells to protect against the degeneration of tufted cells. Several previous reports indicated that astrocytes have the ability to release IL-10 to protect brain tissue when they are stimulated (Mizuno et al., 1994; Sawada et al., 1995; Rasley et al., 2006). The upregulated GFAP mRNA expression shows that astrocytes react to nasal inflammation and also odor deprivation. The reactive astrocytes may release IL-10 to maintain OB homeostasis. Within a damaged nasal cavity, for example, *Staphylococcus aureus* can infiltrate the ONL and GL but not deeper inner layers, possibly because of the actions of activated microglia at the periphery of the OB (Herbert et al., 2012). Activated microglia produce osteopontin, a neuroprotective cytokine, which acts on CD44 expressed by hippocampal neurons and astrocytes following kainic acid–induced excitotoxic hippocampal injury. This signaling is essential for the neuroprotection and remodeling of hippocampal tissue (Hasegawa-Ishii et al., 2011). Thus, the activated microglia in mice with chronic nasal inflammation in the present study may similarly protect the inner layers of the OB (i.e., the MCL, IPL, and GCL) from atrophy.

Although the GL and EPL completely recovered from atrophy only in the presence of odor input, the abatement of nasal inflammation resulted in only partial recovery of OSNs, which occurred regardless of odor input. This suggests that odor stimulation is not necessary for OSNs to regenerate, mature, and project axons, but is required for OB neurons to form and maintain synapses. An intriguing result is that the recovery of OSNs was more efficient in LPS:10w+NC:10w than in LPS:10w+NT:10w. When we examined the expression of OMP, calretinin, and GAP43 in the OE, the number of GAP43-positive and calretinin-positive OSNs increased in NC:3w (Extended Data Fig. 8-1*A*,*B*), and the increase in the number of calretinin–positive OSNs was more prominent on the closed side in NC:10w than on the open side (Extended Data Fig. 8-1*C*,*D*). In the OE, calretinin is expressed by intermediate cells during OSN development (Wei et al., 2013), and we observed a significant increase in the number of calretinin-positive OSNs on the ipsilateral side in LPS:10w+ NC:10w compared with that on the contralateral side and to the ipsilateral side of the OBs in LPS:10w+NT:10w (Extended Data Fig. 8-1*E–G*). This result suggests that NC stimulates the proliferation of basal cells and promotes the turnover of the OSNs, representing a mechanism underlying the better recovery of the OE in LPS:10w+NC:10w than in LPS:10w+NT:10w.

OB atrophy induced by odor deprivation also occurs in neonates. NC in neonatal mice significantly reduces the number of granule and external tufted cells but not mitral cells, which develop earlier (Brunjes, 1985, 1994; Frazier and Brunjes, 1988). Thus, there may be a critical neonatal period during which OSN activity supports the survival of granule and external tufted cells and their integration into the neuronal circuit. Another study in which the nostrils of rats were closed on postnatal day 1 and reopened on postnatal day 20 showed that the OBs recovered from atrophy and that the area of the layer returned to normal (Cummings et al., 1997). However, the external tufted cells were not quantified in that study. Further studies examining the sEPL ratio after naris reopening will help to elucidate the mechanisms underlying OSN activity-dependent OB atrophy and recovery.

Our results suggest that in adults, the dendrites of tufted cells retain plasticity according to OSN activity. In developing neurons, neuronal activity facilitates dendritic stabilization, and the BDNF signaling through TrkB appears to be an important mediator of this effect (Imamura and Greer, 2009). Transgenic mice with lacZ expressed under the control of the BDNF promoter revealed that BDNF is expressed by a subset of interneurons and external tufted cells, but not by mitral cells, in the adult OB (Clevenger et al., 2008). Since TrkB is expressed by both mitral and tufted cells, the differential expression of the BDNF may be a factor underlying their differing dendrite plasticity. In the adult brain, tufted cells are more sensitive to odor inputs and fire in rhythm with respiration (Nagayama et al., 2004; Fukunaga et al., 2012; Igarashi et al., 2012), suggesting that tufted cell circuit is poised to monitor the chemicals and odors in the environment. The high plasticity of tufted cells may be indispensable to monitor the outer chemical environment.

The impact of odor deprivation may not be limited to the OB, because the anterior olfactory nucleus also shrinks (Barbado et al., 2001) and semilunar cells of the piriform cortex undergo apoptosis in response to odor deprivation (López-Mascaraque and Price, 1997; Leung and Wilson, 2003). Further studies are needed to address whether cortical areas and neurons highly vulnerable to the effects of nasal inflammation and/or odor deprivation predominantly receive input from tufted cells, and whether they are associated with the circuitry changes observed in the OB. A greater understanding of the extent to which nasal inflammation and odor deprivation impact neurons will clarify the relationship between the olfactory system and whole brain functions as well as the association between nasal inflammation and neurologic disorders.

## Synthesis

Reviewing Editor: Jörg Schulz, RWTH Aachen University Decisions are customarily a result of the Reviewing Editor and the peer reviewers coming together and discussing their recommendations until a consensus is reached. When revisions are invited, a fact-based synthesis statement explaining their decision and outlining what is needed to prepare a revision will be listed below. The following reviewer(s) agreed to reveal their identity: Kumi Nagamoto-Combs. Note: If this manuscript was transferred from JNeurosci and a decision was made to accept the manuscript without peer review, a brief statement to this effect will instead be what is listed below.

This study addresses potential etiology of brain dysfunction via the olfactory epithelia, the only central nervous system structure that has direct access to the external environment. Chronic or repeated entry of noxious agents that leads to inflammation of olfactory bulbs could be a possible cause of neuroinflammation. The study is carefully performed, and the data presented sufficiently support the authors conclusions. The manuscript may be further improved by addressing the following points, however.

1. The sample sizes are rather small, especially for histological analysis of layer shrinkage. The authors only use 2 sections from each of the 3-4 mice per experimental groups. Although mouse olfactory bulbs are small, more than two 20-μm-thick sections should be acquired and analyzed to improve representation of their findings.

2. It would be more comprehensive to investigate other common proinflammatory cytokines and markers, such as TNFα, IL-6, COX-2, etc. Induction of IL-10 in both LPS and NC is described but not discussed.

3. Discuss or explain why olfactory sensory neurons would recover from LPS-induced inflammation more efficiently with naris closure than no treatment.

4. Figure 7 and 8 compare the effect of LPS {plus minus} NC on specific layers of OB. Although LPS-induced inflammation subsides in LPS + NC group, OB atrophy continues to progress. This may be due to tandem insult to different layers (first to ONL, GL, and EPL by LPS, then to EPL, MCL and GCL by NC). It would be better presented with NC10w data to see if the progressive OB atrophy in LPS+NC represents an additive effect.

5. “Area” or “volume” may be a better term to use than “thickness” when referring to the area measurements of layers in coronal sections. Furthermore, when there are visible differences between medial vs. lateral OB layers, more significant region (side)-specific changes may be obtained if the authors measure the medial and lateral sides individually.

6. Although the authors mentioned the use of Adobe Photoshop and ImageJ software for the the measurements of specified tissue areas, the detailed process was not explicitly explained. This should be included.

7. The authors’ extended data should be included in the main body of the manuscript since they use the data to support their arguments.

## Author Response

March 7, 2020

Dear Reviewer and Editor,

We very much appreciate your insightful review of our paper entitled “Differential effects of nasal inflammation and odor deprivation on layer-specific degeneration of the mouse olfactory bulb”. The comments and recommendations from the reviewer and editor were very constructive, and we have worked hard to ensure that they have all been fully addressed. We hope that you will agree that the incorporation of these into a revised manuscript is appropriate and results in a much stronger submission to eNeuro. The responses to each of the comments are detailed below:

Synthesis Statement for Author:

This study addresses potential etiology of brain dysfunction via the olfactory epithelia, the only central nervous system structure that has direct access to the external environment. Chronic or repeated entry of noxious agents that leads to inflammation of olfactory bulbs could be a possible cause of neuroinflammation. The study is carefully performed, and the data presented sufficiently support the authors conclusions. The manuscript may be further improved by addressing the following points, however.

Response: Thank you very much for the positive comments.

1. The sample sizes are rather small, especially for histological analysis of layer shrinkage. The authors only use 2 sections from each of the 3-4 mice per experimental groups. Although mouse olfactory bulbs are small, more than two 20-μm-thick sections should be acquired and analyzed to improve representation of their findings.

Response: We appreciate the reviewer’s comment. As he/she pointed, the length of the olfactory bulbs in adult male mice is ∼2.6 mm from the rostral to caudal ends. To compare the relative layer sizes among mice at the same rostro-caudal level and since the rostral parts of the olfactory bulb are much smaller and caudal parts have much thinner olfactory nerve layer, we restricted our histological analyses to the “middle olfactory bulb”, where the area of olfactory bulb on the contralateral side is 3-4 mm2 and the area of the olfactory nerve layer on the contralateral side is {greater than or equal to}0.5 mm2. To improve representation of our findings, we added one section per each mouse for the analysis (three sections from each mouse) and replaced the associated graphs in Figures 3 and 8 to new Figures 3 and 10, respectively. We also modified the associated sentences in the Method and Results sections of the revised manuscript.

2. It would be more comprehensive to investigate other common proinflammatory cytokines and markers, such as TNFα, IL-6, COX-2, etc. Induction of IL-10 in both LPS and NC is described but not discussed.

Response: According to the reviewer’s comments, we performed qPCR for TNFα, IL-6, and COX-2 as common pro-inflammatory factors and TGFβ and BDNF as common anti-inflammatory factors using the ipsilateral side of the olfactory bulbs from saline-, LPS-, and NC-treated mice. The results indicated that the expression of TNFα was higher in LPS:10w mice than in saline:10w and NC:10w mice. The expression levels of the other molecules (IL-6, COX-2, TGFβ or BDNF) did not differ. These new data support our conclusion that chronic nasal inflammation, but not naris closure, caused neuroinflammation in the olfactory bulb. We added these data in a new Figure 7 and in the Results section in the revised manuscript.

In NC mice, glial cells did not show obvious morphological changes. However, our qPCR experiments showed increased expression of GFAP mRNA not only in LPS mice but also in NC mice (new Figure 7). This suggests that NC affects astrocyte activity and is consistent with the elevation of IL-10 mRNA in the OBs of NC mice since several previous reports indicated that astrocytes have the ability to release IL-10 when they are stimulated to help resolve tissue damage (Mizuno et al. Biochem Biophys Res Commun, 1994. 205(3):1907-1915; Sawada et al. Int J Dev Neurosci, 1995. 13(3-4):253-264; Rasley et al. GLIA, 2006. 53:583-592). We speculate that astrocytes react to atrophy and release IL-10 in the OBs of NC mice, although the possibility that astrocyte reactivity induces EPL shrinkage cannot be discounted. We now address this in the Discussion of the revised manuscript.

3. Discuss or explain why olfactory sensory neurons would recover from LPS-induced inflammation more efficiently with naris closure than no treatment.

Response: Thank you very much for pointing this out. It was a very intriguing result that the olfactory sensory neurons (OSNs) recovered more efficiently in LPS:10w+NC:10w mice than in those with LPS:10w+NT:10w. To see the effect of NC in the OSNs, we examined the expression of OMP (mature OSN), calretinin (intermediate OSN), and GAP43 (immature OSN) in the OE of NC:3w and NC:10w mice. Interestingly, we found that the number of GAP43-positive and calretinin-positive OSNs increased on the closed side compared with that on the open side in NC:3w mice. The increase in calretinin-positive OSNs was retained until 10 weeks. We also observed a significant increase in calretinin-positive intermediate OSNs in the ipsilateral OBs from LPS:10w+NC:10w mice compared to that on the contralateral side as well as the ipsilateral OBs from LPS:10w and LPS:10w+NT:10w mice. This observation suggests that NC stimulates the proliferation of basal cells and promotes the turnover of OSNs as potential underlying mechanism for the better recovery of the olfactory epithelium in LPS:10w+NC:10w mice than in those with LPS:10w+NT:10w. Although the regeneration of OSNs is not a focus of this study, which examined the differential effects between olfactory inflammation and odor deprivation, we added this interesting speculation to the Discussion of the revised manuscript and Figure 8-1.

4. Figure 7 and 8 compare the effect of LPS {plus minus} NC on specific layers of OB. Although LPS-induced inflammation subsides in LPS + NC group, OB atrophy continues to progress. This may be due to tandem insult to different layers (first to ONL, GL, and EPL by LPS, then to EPL, MCL and GCL by NC). It would be better presented with NC10w data to see if the progressive OB atrophy in LPS+NC represents an additive effect.

Response: As the reviewer commented, we considered that the LPS+NC group suffered from tandem insult of LPS plus NC. When we statistically analyzed the layer areas among saline, LPS, NC, LPS+NC, and LPS+NT groups, we found that all layers were damaged in the LPS+NC group. The areas of the ONL and GL were smaller than in NC mice, and EPL, MCL+IPL, and GCL were smaller than in LPS mice, indicating that the LPS+NC group was affected by both LPS and NC. Therefore, we include this mention in the Results of the revised manuscript. However, the purpose of comparing the effect of LPS {plus minus} NC was to examine whether odor input is necessary for recovery from LPS insult. Thus, we have decided not to include the data comparing NC:10w with LPS:10w, LPS:10w+NT:10w, and LPS:10w+NC:10w in the figure (see new Figure 10). We hope the editor and reviewers agree with our decision.

5. “Area” or “volume” may be a better term to use than “thickness” when referring to the area measurements of layers in coronal sections. Furthermore, when there are visible differences between medial vs. lateral OB layers, more significant region (side)-specific changes may be obtained if the authors measure the medial and lateral sides individually.

Response: We thank the reviewer for his/her comment. We have changed “thickness” to “area” in the revised manuscript.

A previous paper showed that layers on the lateral side of the olfactory bulb are more vulnerable to nasal administration of LPS than those on the medial side at 3 weeks (Hasegawa-Ishii et al. Sci Rep, 2017, 7(1):11605). However, the medial side is more comparably affected with longer duration of LPS administration (Hasegawa-Ishii et al. J. Allergy Clin Immunol, 2019,143(3):978-989). Furthermore, when we separated the olfactory bulb layers in NC mice into dorsomedial and ventrolateral regions according to positive and negative NQO-1 expression, respectively, we did not observe any prominent differences in shrinkage. Thus, we believe that the data obtained from both the medial and lateral sides of the OB are sufficient to reveal the differential effects of olfactory inflammation and naris closure on the OB. We hope the editor and reviewers agree.

6. Although the authors mentioned the use of Adobe Photoshop and ImageJ software for the measurements of specified tissue areas, the detailed process was not explicitly explained. This should be included.

Response: We sincerely apologize for the unclear description of histological methods. We have included detailed explanations in the Methods section of the revised manuscript.

7. The authors’ extended data should be included in the main body of the manuscript since they use the data to support their arguments.

Response: We appreciate the reviewer’s comments. Accordingly, we moved the extended data to the main figures. The extended data (1-1, 3-1, and 5-1) are now included in Figures 1, 4, and 7, respectively.
