# Supplementary figures and images for: Differential Effects of Nasal Inflammation and Odor Deprivation on Layer-Specific Degeneration of the Mouse Olfactory Bulb
Source: eNeuro. 2020 Apr 2;7(2):ENEURO.0403-19.2020. doi: 10.1523/ENEURO.0403-19.2020 (PMC7168263; doi:10.1523/ENEURO.0403-19.2020)

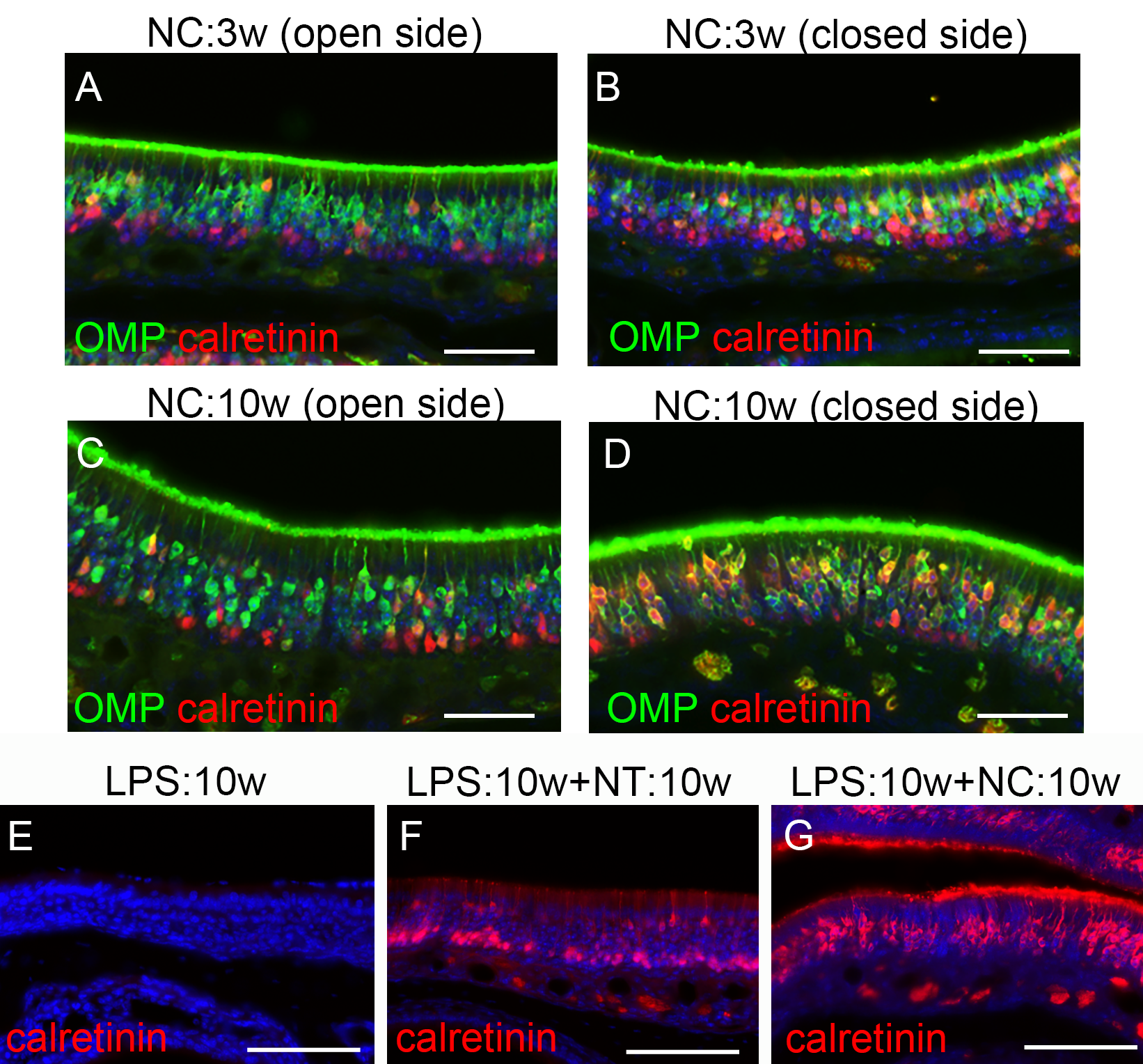

Supplement: Extended Data Figure 8-1 — Increased number of calretinin-positive OSNs in the closed side of NC. A–D, Coronal sections of the OE stained for OMP (green), calretinin (red), and nuclei (DAPI, blue). Calretinin-immunopositive intermediate OSNs increased in the closed side of NC:3w and NC:10w compared with that in the open side. Note that the number of OMP- and calretinin-double immunopositive cells increase in the closed side of NC mice. E–G, Coronal sections of the OE stained for calretinin (red) and nuclei (DAPI, blue). Calretinin-immunopositive OSNs are lost in LPS:10w (E) and regenerated in a patchy manner in the presence (F) and absence (G) of odor input. Note that calretinin-immunopositive OSNs remarkably increased in number in LPS:10w+NC:10w. Download Figure 8-1, TIF file. [file enu-eN-NWR-0403-19-s02.tif]
